# An *in vivo* evaluation of the safety and efficacy of using decellularized bovine parietal peritoneum membranes as dural substitutes

**DOI:** 10.3389/fsurg.2024.1432029

**Published:** 2024-12-06

**Authors:** Aidos Doskaliyev, Vyacheslav Ogay, Islambek Mussabekov, Muratbek Satov, Berik Zhetpisbayev, Khalit Mustafin, Xeniya Bobrova, Raushan Auezova, Serik Akshulakov

**Affiliations:** ^1^ Department of Science and Strategy, National Center for Neurosurgery, Astana, Kazakhstan; ^2^ Stem Cell Laboratory, National Center for Biotechnology, Astana, Kazakhstan; ^3^ Department of Education, National Center for Neurosurgery, Astana, Kazakhstan; ^4^ Department of Pathological Anatomy, National Center for Neurosurgery, Astana, Kazakhstan; ^5^ Department of Neurosurgery and Neurology, National Center for Neurosurgery, Astana, Kazakhstan; ^6^ Department of Research Management, National Center for Neurosurgery, Astana, Kazakhstan; ^7^ National Center for Neurosurgery, Astana, Kazakhstan

**Keywords:** bovine parietal peritoneum, dural substitute, duraplasty, dura matter, regeneration

## Abstract

**Purpose:**

The reconstruction of dura matter is a challenging problem for neurosurgeons. A number of materials for dural reconstruction have recently been developed, but some of them have poor biocompatibility, poor mechanical properties, and adverse effects. Bovine parietal peritoneum is a promising natural material for regenerative medicine and reconstructive surgery. In this study, we conducted an *in vivo* evaluation of the safety and efficacy of using decellularized bovine peritoneum membranes (BPMs) as natural dural substitutes in a rabbit model.

**Methods:**

The dural defects in mature New Zealand rabbits were studied. A BPM was sutured on the dural defect area of each animal. Autologous periosteum and collagen membranes (Lyoplant®) were used to facilitate a comparison with the BPMs. ELISA, histomorphological analysis, and hematological analysis were carried out to examine the safety and efficacy of using BPMs as dural substitutes.

**Results:**

Our results showed that the BPMs demonstrated a deterioration rate that is suitable for gathering newly formed meningothelial tissue. The thickness and density of BPM fibers prevents resorption in the first few days after use as a plastic material, and the regeneration of the dura mater does not occur at an accelerated pace, meaning that the gradual formation of fibrous tissue prevents adhesion to the brain surface. It was observed that the BPM can integrate with the adjacent tissue to repair dural defects. Moreover, the transplantation of BPMs did not cause significant adverse effects or immunological responses, indicating the safety and good biocompatibility of the BPM.

**Conclusion:**

Thus, our *in vivo* study in a rabbit model showed that decellularized BPMs may represent a biocompatible natural material that can be used in cases requiring dura matter repair without significant adverse effects.

## 1 Introduction

Restoring lost or damaged tissue is one of the main tasks of modern medicine and is especially important in the treatment of wounds. Various factors influence wound healing, and poor healing results have many negative effects on the body. Wounds that heal very slowly or do not heal at all are an important cause of morbidity and mortality in patients and pose a challenge for researchers and clinicians (1).

In the predominant neurosurgical operations, an obligatory step is the dissection of the dura mater (DM) with its subsequent restoration. In some cases, the neurosurgeon often faces the problem of DM closure when the existing DM is not enough to completely close the defect. In turn, dural defects represent a significant problem both for the patient and for the surgeon, since incomplete closure can lead to cerebrospinal fluid leakage and related complications (2, 3).

Dural defects can be reconstructed using a number of artificial or natural materials, where the graft closing the defect acts as a matrix that fills the wound gap (or tissue defect) and ensures the growth of various types of cells and connective tissues (4, 5).

In neurosurgical practice, autologous periostea (APs) consisting of collagen membranes such as the periosteum of the skull, fascia of the temporal muscle, and wide fascia of the thigh are usually used as substitutes for DM, since they do not cause an immunological or severe inflammatory response (6). Among the above, the periosteum has the highest density of elastic fibers and is an ideal transplant from the point of view of compatibility with the host, and it also has a low complication rate and ensures the tightness of DM (7–10).

Synthetic substitutes, which have a number of advantages, are also used; however, they are not widely used in clinical practice due to the specific disadvantages associated with their use, such as pronounced aseptic inflammation in the surrounding tissues with the formation of a rough connective tissue scar; their weak resistance to the infectious process, which limits their application in neurosurgical injuries; and the violation of sealing during the response of the brain in the form of cerebral edema in the postoperative period (8, 11, 12).

All biological tissues contain non-cellular components that form well-organized networks which comprise the extracellular matrix (extracellular matrix). Extracellular matrices not only provide the physical scaffolds into which cells integrate but also regulate many cellular processes, including growth, migration, differentiation, survival, homeostasis, and morphogenesis. The composition and specific structures of the extracellular matrix vary in different tissues, though the main components are highly acidic and hydrated molecules such as collagens, elastin, fibronectin, laminins, glycoproteins, proteoglycans, and glycosaminoglycans (13).

Because native DM is primary composed of type I collagen, purified and reconstituted fibrous type I collagen-based dural substitutes could meet the biological safety requirements and have the potential to regenerate the dural tissue via an appropriate structural design. In fully degradable dural substitutes based on natural materials, many products are based on type I collagen derived from different animal tissues. Existing data predominantly suggest that collagen-based dural substitutes are acceptable for clinical applications, with multiple studies reporting satisfactory results.

The National Center for Biotechnology developed a method for treating burn wounds using a relatively inexpensive wound dressing that is obtained from a bovine's parietal peritoneum (14). They found an efficient technological setup for decellularizing BPP and sterilizing it with radiation; also, preclinical testing showed safety of the material obtained from a bovine's parietal peritoneum. Decellularized bovine peritoneum wound dressings demonstrated good adhesion to the wound bed, reducing pain, the number of dressing changes, and fluid loss in a clinical trial of patients with IIA grade burn injuries (15, 16).

The study's novelty arises from its investigation of a new biomaterial—bovine peritoneum membrane (BPM) for DM grafting as a natural dural substitute on a rabbit model to evaluate the *in vivo* safety and efficacy in comparison with autologous periostea and pure collagen membranes (Lyoplant®) produced from lyophilized bovine pericardium.

## 2 Materials and methods

### 2.1 Animals

Male New Zealand rabbits (10–12 weeks old) were purchased from SPF-vivarium at M. Aykimbayev's National Scientific Center of Especially Dangerous Infections (Almaty, Kazakhstan). The rabbits were held in large cages (Techiplast, Italy) at a temperature of 23°C and a relative humidity of 60%. All experimental animals had *ad libitum* access to food and water. All procedures involving laboratory animals and their care were carried out in full compliance with the international principles of the European Convention for the Protection of Vertebrate Animals used for experimental and other scientific purposes and were approved by the Local Ethics Committee for Animal Use in the National Center for Biotechnology (Kazakhstan).

### 2.2 Preparation of decellularized bovine parietal peritoneum

The bovine peritoneum membranes (BPMs) were processed according to the detergent enzymatic method (17). Briefly, the BPMs were washed in phosphate-buffered saline (PBS) containing 1% povidone-iodine (PI). Afterwards, the BPMs were washed twice in distilled water (containing 1% PI) and subsequently washed twice in Milli-Q water containing 1% penicillin/streptomycin/amphotericin B. In order to remove cell debris, the BPMs were treated with 4% sodium deoxycholate for 4 h at room temperature and washed twice in distilled water. This process was repeated twice. To completely remove cell DNA, the BPMs were incubated with DNase I (2,000 KU) in 1 M NaCl in a shaker/incubator for 3 h at 37°C and then washed three times in distilled water. The decellularized BPMs were packed in blister packs and sterilized with β-radiation at a dose of 16 kGy.

### 2.3 DNA quantification

DNA quantification was conducted on both native (*n* = 6) and decellularized (*n* = 6) BPM. Previous research suggests that a dsDNA content of less than 50 ng/mg of matrix is an effective indicator of successful decellularization (18). The BPM samples were weighed, then digested overnight at 55°C using Proteinase K, PBS, and lysis buffer (0.1 M Tris pH 8, 0.2 M NaCl, 5 mM EDTA, 0.4% SDS). Following digestion, DNA was extracted from the entire BPM using a phenol/chloroform/isoamyl alcohol-based protocol. The amount of DNA per milligram of dry tissue was quantified with the DNA Quantitation Kit (Sigma-Aldrich, USA).

### 2.4 Tissue processing and histological scoring

The tissue samples were fixed in 10% neutral-buffered formalin. Next, the samples were sequentially dehydrated in 70%, 95%, 95%, 100%, 100% ethyl alcohol and immersed in xylene. Then, the samples were infiltrated with paraffin, embedded into paraffin blocks, and cut into 5 μm sections. Before staining, the sections were treated with xylene and sequentially rehydrated in 100%, 100%, 95%, 95%, and 70% ethyl alcohol and distilled water in order to remove the paraffin. The sections from each sample were stained with hematoxylin and eosin (H&E) and Masson's trichrome (TRI). The stained sections were visualized using a digital slide scanner 3DHISTECH PANNORAMIC Midi (Carl Zeiss, Germany). All acquired images were processed using Image J software (NIH, USA).

### 2.5 Transmission electron microscopy

The decellularized BPM samples were fixed with 2.5% glutaraldehyde in PBS (pH 7.3) and post-fixed with1% OsO_4_. After dehydration with ethanol and propylene oxide, the samples were embedded in Epon 812 resin. Ultrathin sections were prepared using an ultramicrotome (UC5; Leica, Wetzlar, Germany). Ultrathin sections were collected on large-scale copper grids, contrasted using 2% uranyl acetate and Reynolds' lead citrate, and examined using a transmission electron microscope (Libra-120, Carl Zeiss, Germany) at an accelerating voltage of 80 kV, and images were obtained using a digital camera (Carl Zeiss, Germany).

### 2.6 Enzyme-linked immunosorbent assay

Peripheral blood was collected from the ear veins of the rabbits before surgery as well as at 7, 14, 30, and 60 days after surgery. Serum was obtained from the peripheral blood by centrifugation for 5 min at 400 g. The concentrations of CRP and TNF-α were measured using enzyme-linked immunosorbent assay (ELISA) kits (Abcam, UK) according to the manufacturer's instructions.

### 2.7 Surgery procedure

The rabbits were anesthetized with 40 mg/kg of ketamine hydrochloride, which was administered intramuscularly. Before surgery, the wool was removed from the heads of the rabbits using clippers, and then the shaved area was treated with a povidone-iodine solution. To prevent post-operative complications, the rabbits received intramuscular injections of 0.01 mg/kg enrofloxacin before surgery. For local anesthesia, the rabbits received injections of 2% lidocaine hydrochloride in the surgical incision area.

**Figure 1:**
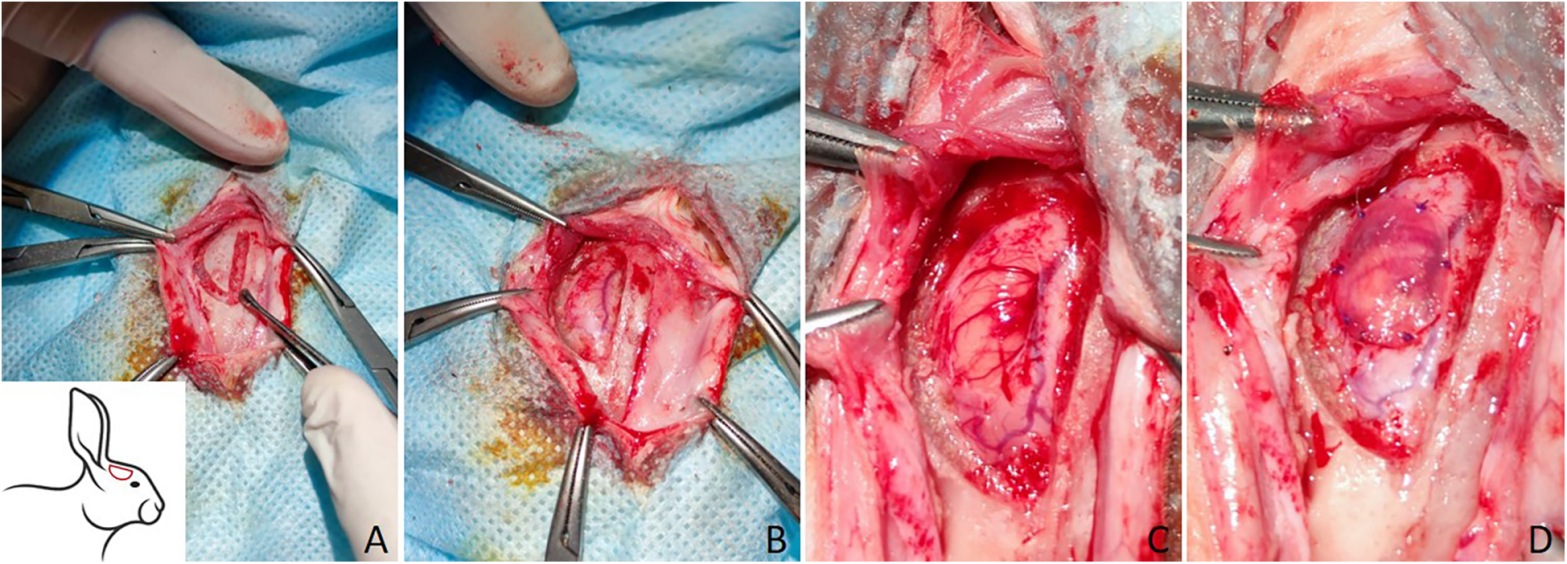
The surgical procedures of trepanation of the rabbit skull and transplantation of the materials for the plastic surgery of DM. **(A)** Trepanation of the skull using an electric drill spherical grooved and diamond tips, the trepanation area is depicted schematically in the lower left corner; **(B)** a burr hole after cranial trepanation; **(C)** dura matter defect; **(D)** the plastic material is fixed with sutures.

The different stages of the surgical procedures are presented in Figure 1. A 4 cm-long skin incision was made longitudinally along the midline from the junction of the nasal and frontal bones at the level of the anterior upper corner of the eyes to the interparietal bone at the level of the base of the auricles. The edges of the surgical wound were fixed with clamps and folded back, and sterile drapes were placed around the wound. The muscles, as well as the periosteum, were separated from the surface of the skull bone layer by layer using a dissector. Cranial trepanation in the right fronto-parietal–temporal bone was performed using an electric drill with spherical grooves and diamond tips. During cranial trepanation, the animal tissues were irrigated with a sterile saline to prevent the adjacent tissues from suffering thermal burns.

The formed bone flap was separated from DM using a dissector and removed. The surface of the DM was cleaned to remove bone chips and subsequently washed with warm saline. Under a stereomicroscope, a 4 × 6 mm DM defect was formed. Next, the materials were prepared for the plasty of the DM defect.

The experimental animals in group 1were transplanted with a decellularized BPM (*n* = 7). The experimental animals in group 2 were transplanted with autologous periostea (APs) (*n* = 7). The experimental animals in group 3 were transplanted with Lyoplant® collagen membranes (Aesculap AG, Germany) (*n* = 7). The repair materials were fixed with 6/0 absorbable sutures after careful the hemostasis and lavaging of the cortical surface. Layer-by-layer wound suturing was carried out using an absorbable suture material with a diameter of 4/0. The wounds were treated using povidone-iodine solution.

After the observation period, the animals were sacrificed with a minimum of physical and mental suffering by trained personnel. The rabbit was placed in a special transparent chamber with a supply of carbon dioxide. Animals were left in the chamber for an additional five minutes when there was no longer any visible movement.

The implant, surrounding DM, brain, and soft tissues were all collected in order to evaluate the implant's integrity, degree of inflammation, degree of adhesion to brain tissue, and the presence of cerebrospinal fluid leakage.

### 2.8 Statistical analysis

All obtained data are presented as mean ± SD. Statistical significance was calculated using a one-way ANOVA followed by Bonferroni's multiple comparison tests. *P* < 0.05 was considered the threshold for statistical significance. Our statistical analysis was conducted using Statistica 6.0 software (StatSoft, Tulsa, OK, USA).

## 3 Results

### 3.1 Characterization of decellularized BPM

**Figure 2:**
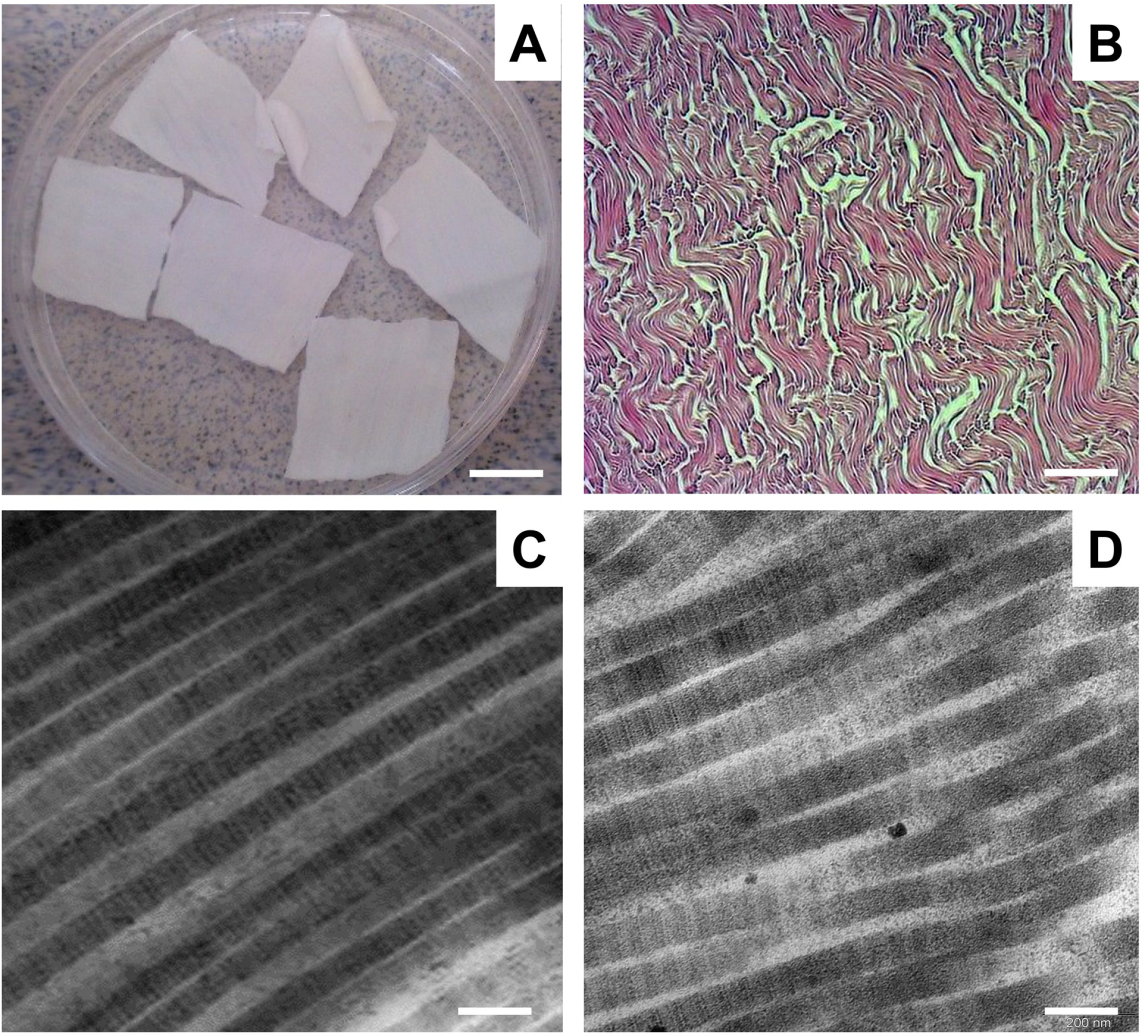
Representative gross and microscopic images of decellularized BPM. **(A)** Gross images of decellularized BPM. Scale bar 100 mm. **(B)** Representative image of decellularized BPM sectioned and stained with H&E. Scale bar 200 µm. **(C)** Representative transmission electron microscopic (TEM) image of collagen fibrils of BPM before decellularization. **(D)** TEM micrograph of collagen fibrils in decellularized BPM. Scale bar 200 nm.

To obtain a collagen–elastin scaffold from the BPMs, we used a detergent–enzymatic method that was previously proposed by Keane and colleagues (19, 20). To obtain a cellular collagen–elastin scaffold from the BPMs, we used two cycles of detergent–enzymatic treatment. Each cycle of BPM decellularization consisted of three main steps: (1) osmotic cell lysis and the removal of intracellular components using ultrapure Milli-Q water, (2) treatment with 4% sodium deoxycholate for 4 h to remove the cell membranes and cytoplasmic proteins of the cells, and (3) enzymatic treatment with DNase-I in order to digest the DNA released during cell lysis. Representative gross and microscopic images of decellularized BPMs are shown in Figure 2.

Microscopic assessment by H&E staining showed that the decellularized BPMs did not contain any cells and consisted of connective tissue fibers, predominantly collagen fibers (Figure 2B). Electron microscopy revealed that the collagen fibrils were without visible ultrastructure changes after the decellularization of the BPMs (Figure 2C).

### 3.2 DNA quantification

**Figure 3:**
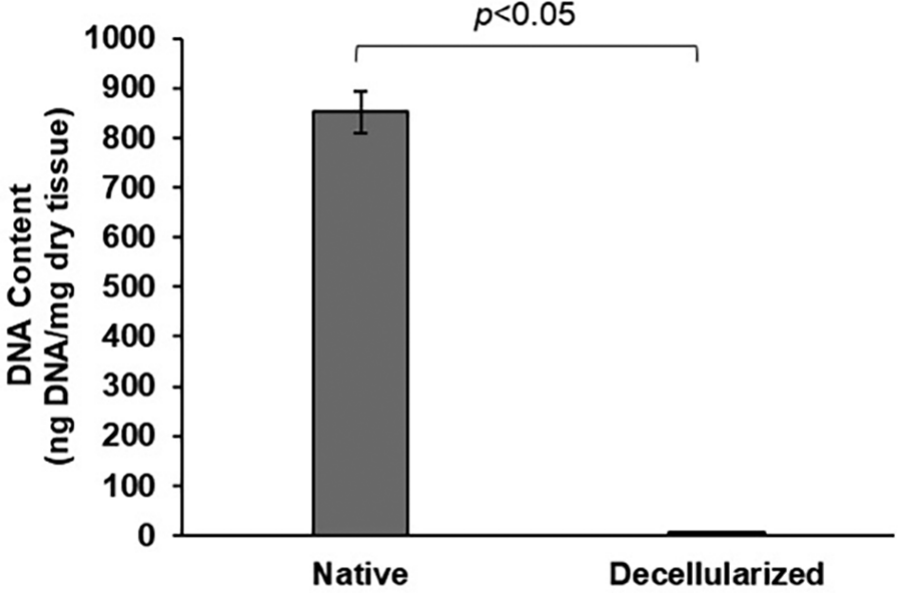
DNA quantification of native and decellularized BPM samples.

After three cycles of decellularization, DNA quantification revealed that decellularized BPM had a DNA content of 5.2 ng/mg of dry tissue, compared to 851.3 ng/mg in native BPM (Figure 3). It indicates that only 0.61% of the original DNA was retained in the decellularized samples. The DNA content in the decellularized tissue sample was significantly lower than that in native BPM (*p* < 0.05).

### 3.3 Histological examination

**Figure 4:**
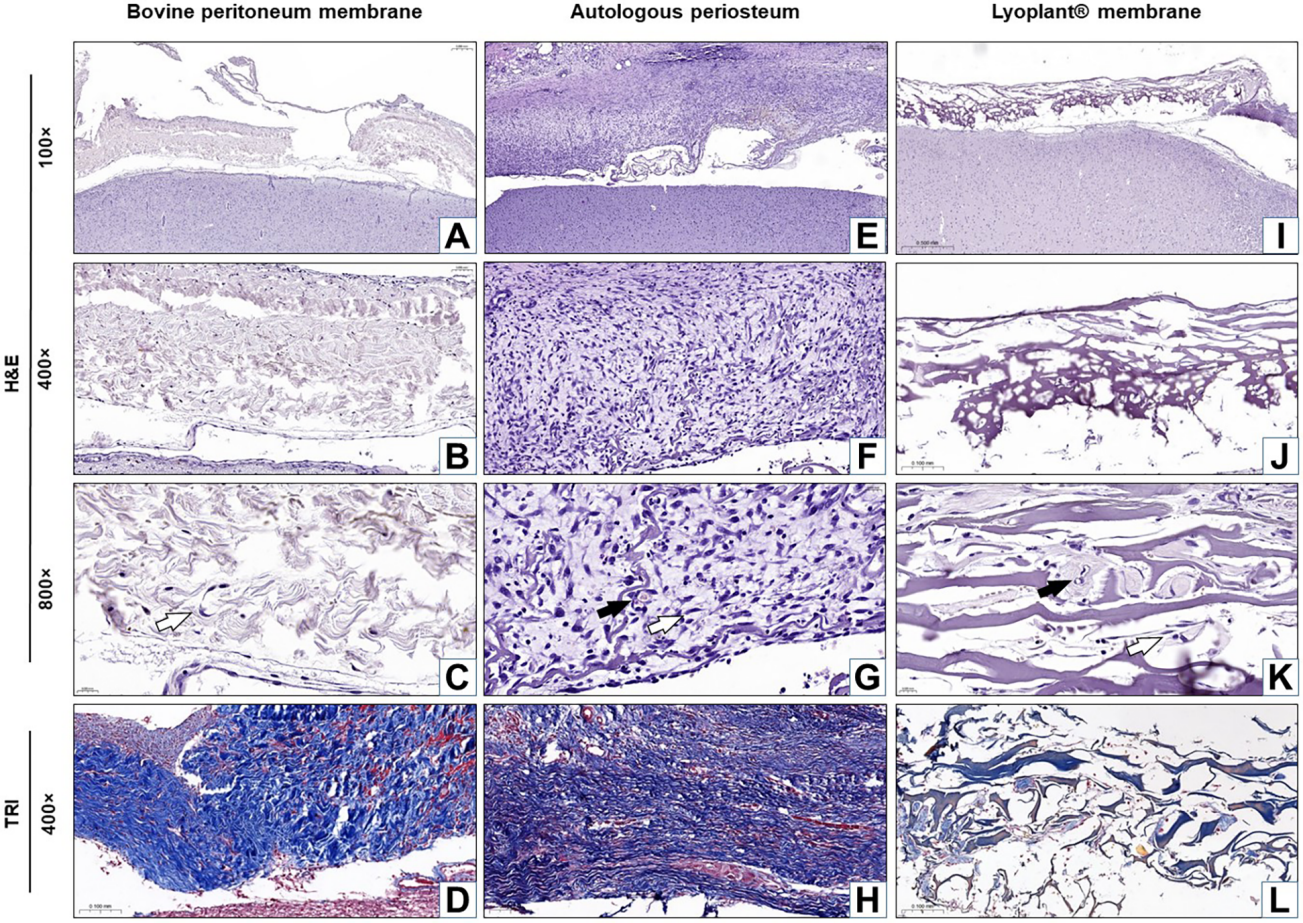
Histological analysis of the implanted dura matter substitutes at day 7 after plastic surgery. Arrows show fibroblasts in the connective tissue membranes 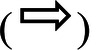 and blood vessels 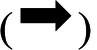. **(A–C**,**E–G**,**I–K)** H&E staining; **(D**,**H**,**L)** Masson's trichrome (TRI) staining.

**Figure 5:**
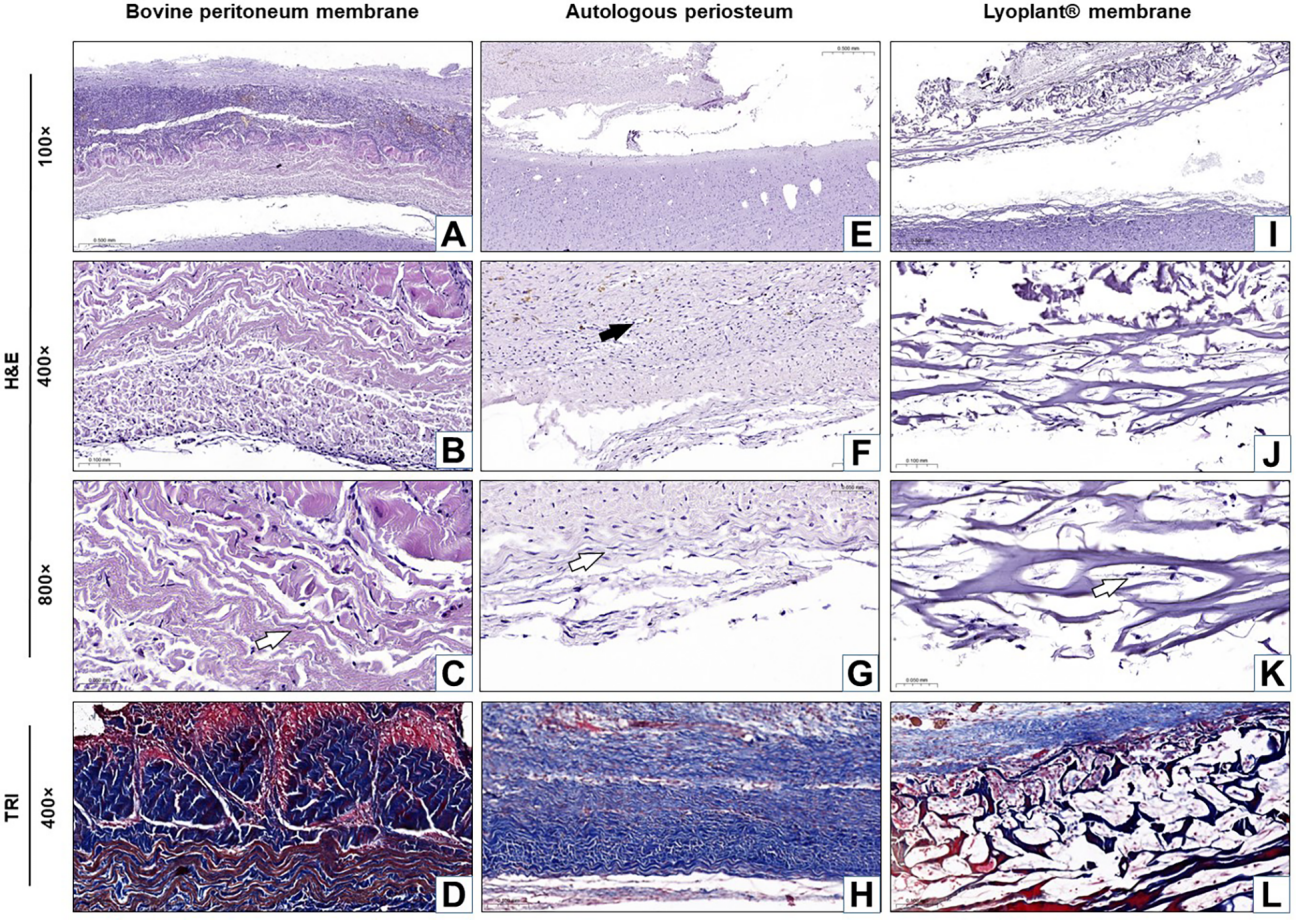
Histological analysis of the implanted dura matter substitutes at day 21 after plastic surgery. Arrows show fibroblasts in the connective tissue membranes 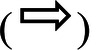 and blood vessels 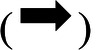. **(A–C**,**E–G**,**I–K)** H&E staining; **(D**,**H**,**L)** Masson's trichrome (TRI) staining.

**Figure 6:**
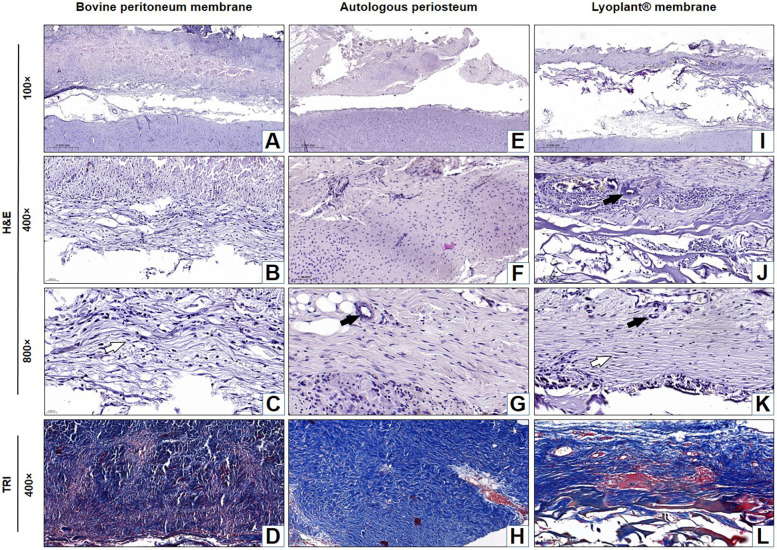
Histological analysis of the implanted dura matter substitutes at day 63 after plastic surgery. Arrows show fibroblasts in the connective tissue membranes 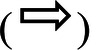 and blood vessels 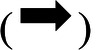. **(A–C**,**E–G**,**I–K)** H&E staining; **(D**,**H**,**L)** Masson's trichrome (TRI) staining.

The results of our histological examination of the implanted dural substitutes (decellularized BPMs, APs, and Lyoplant®) after H&E and Masson's trichrome staining at day 7, day 21, and day 63 are shown in Figures 4–6, respectively.

**Figure 7:**
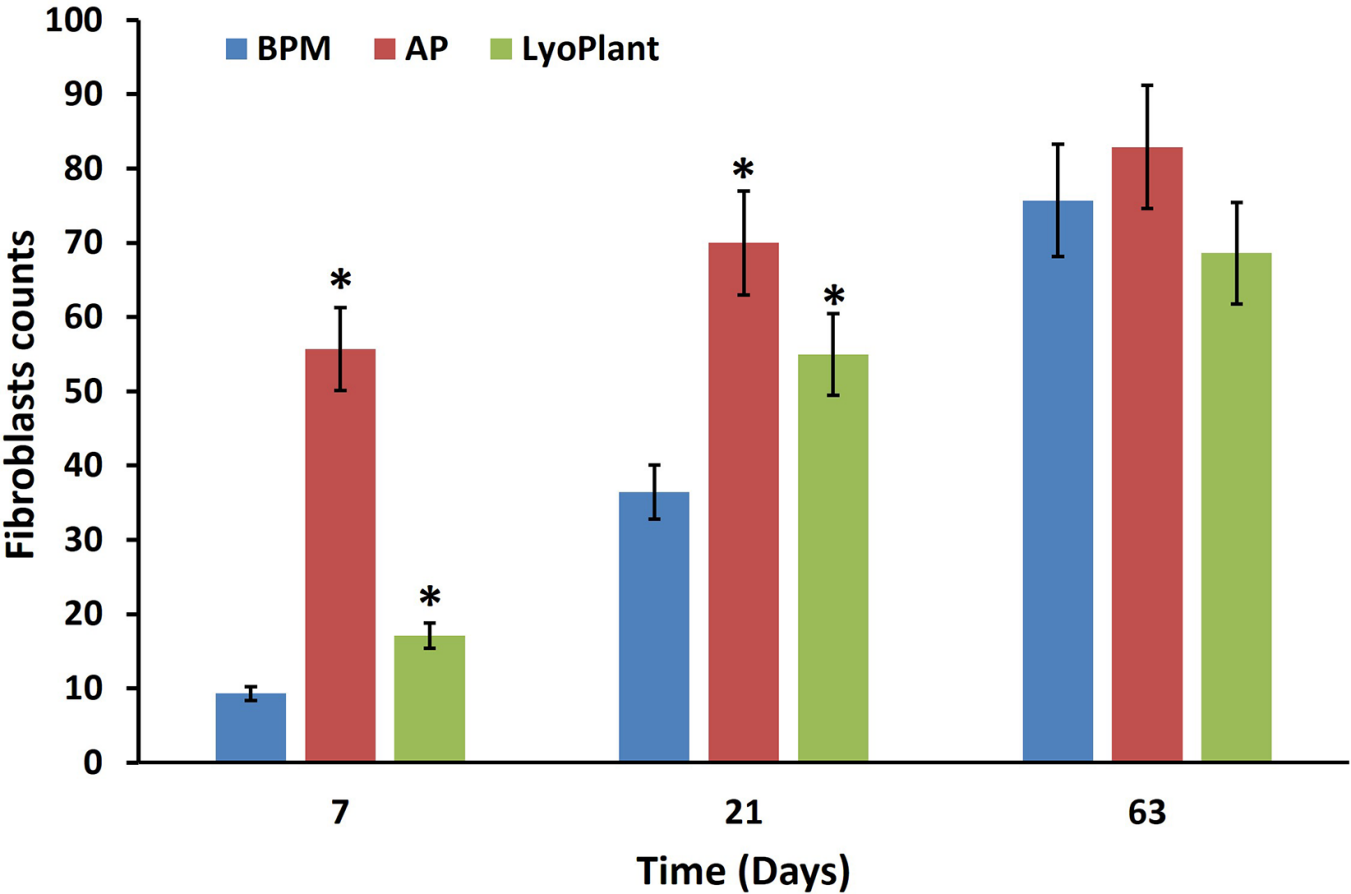
Number of infiltrated fibroblasts in BPM, autologous periosteum (AP) and collagen membrane lyoplant®, data are represented as the mean ± SD, (*n* = 7), *p < 0.05 compared to BPM group.

The average number of fibroblast cells on H&E stained preparations after the implantation of BPM, autologous periostea, and Lyoplant® collagen membranes showed a steady increase in fibroblasts in BPM and Lyoplant® collagen membranes from day 7 to day 63. In contrast to autologous periostea had a higher number of fibroblasts as early on day 7. The quantity of fibroblasts in each of the three materials had been approximately similar on day 63 (Figure 7).

For the decellularized BPM group, the results showed pronounced cellular responses at the border with DM due to the formation of maturing granulation tissue at 7 days after surgical implantation (Figures 4A–D). The cellular composition was represented by inflammatory cells (neutrophils, eosinophils lymphocytes, plasma cells, and macrophages) and fibroblasts (Figures 4B,C). The predominance of inflammatory cells over fibroblasts was determined. No fibrous adhesions were found between the decellularized BPM and the cerebral cortex.

At 21 days after the implantation of the decellularized BPMs, histological examination revealed inflammatory reaction regression, as well as fibroblast (Figure 7) and meningothelial cell infiltration in the extracellular matrix of the decellularized BPMs (Figures 5A–D). At the same time, fibroblasts were more predominant than the inflammatory cells (Figure 7). The implanted BPMs were partially degraded. No fibrous adhesions were found between the decellularized BPM and the cerebral cortex.

After 63 days, there was complete fusion between the decellularized BPMs and DM. The fibers in the BPMs became degraded throughout and were subsequently replaced by connective tissues and meningothelial and fibroblast-like cells with differentiated, newly formed vessels (Figures 6A–D). There was a regression of the inflammatory process with the absence of leukocytes. Inflammatory cell infiltrates were represented by single lymphocytes. No pathological changes were found in the adjacent cerebral cortex.

In the autologous periosteum (AP) group, histological analysis showed the presence of a large number of young fibroblasts in the connective tissue membranes (Figure 7) and blood vessels at the boundary with DM at 7 days after AP implantation (Figures 4E–H). The newly formed connective tissue fibers were thin and slightly crimped and did not have a definite spatial orientation (Figure 4H). The newly formed blood vessels looked like acellular gaps and “vascular buds”. The inflammatory response was characterized by minor neutrophil–leukocyte infiltrates (Figures 4F,G). There were no fibrous adhesions, and pathological changes were found between the AP and the cerebral cortex.

After 21 days of implantation, we found that the inflammatory response had regressed, fibroblasts became mature, connective tissue fibers became denser, and proliferated meningothelial cells appeared at the border with DM (Figures 5E–H). No fibrous adhesions were found between the AP and the cerebral cortex.

At day 63, the AP's complete fusion with DM was observed. There were no inflammatory reactions, and no fibrous adhesions were found between the AP and the cerebral cortex (Figures 6E–H). Fibrous tissue formed mostly above the implants, and compaction of DM was noted over the entire surface of the skull defect. Cerebrospinal fluid leakage or fistula formation were not observed in any experimental animal. The fibrous tissue that grew into the implant and covered the open surface of DM did not adhere to the skin and subcutaneous fat.

For the Lyoplant® dural substitute group, histological examination at 7 days after surgery showed a less pronounced inflammatory response at the border with DM compared to the decellularized BPMs and APs (Figures 4I–L). The formation of new vessels was determined based on the form of acellular gaps and “vascular buds” and the loosening of the collagen fibers of the decellularized BPMs, focusing on their lysis (melting) and expansion into the spaces of the collagen framework (Figures 4J,K). No fibrous adhesions were found between the Lyoplant® and the cerebral cortex. No pathological changes were found in the adjacent cerebral cortex.

At 21 days after the implantation of the Lyoplant®, inflammatory response regression was observed (Figures 5I–L), along with the proliferation of fibroblasts with newly formed connective tissue fibers and meningothelial cells, both of which penetrated into the spaces between the collagen fibers (Figures 5J,K). Fibroblasts, meningothelial cells, and connective tissue fibers replaced half of the area of the Lyoplant® dural substitute (Figure 7). The collagen fibers were also loosened, and the foci of their fragmentation appeared (Figure 5L). No fibrous adhesions were found between the Lyoplant® and the cerebral cortex.

At 63 days after implantation, the artificial DM “Lyoplant®” completely fused with DM (Figures 6I–L). The boundary between the Lyoplant® dural substitute and DM was not clear. To a large extent, the Lyoplant® dural substitute was immersed in the connective tissue framework, the fibers were completely degraded, and the spaces between the collagen fibers were often occupied by connective tissues with newly formed vessels and meningothelial and fibroblast-like cells (Figures 6J,K). Inflammatory infiltrates were represented by single lymphocytes and macrophages. No fibrous adhesions were found between the Lyoplant® and the cerebral cortex.

### 3.4 Immunological response

**Figure 8:**
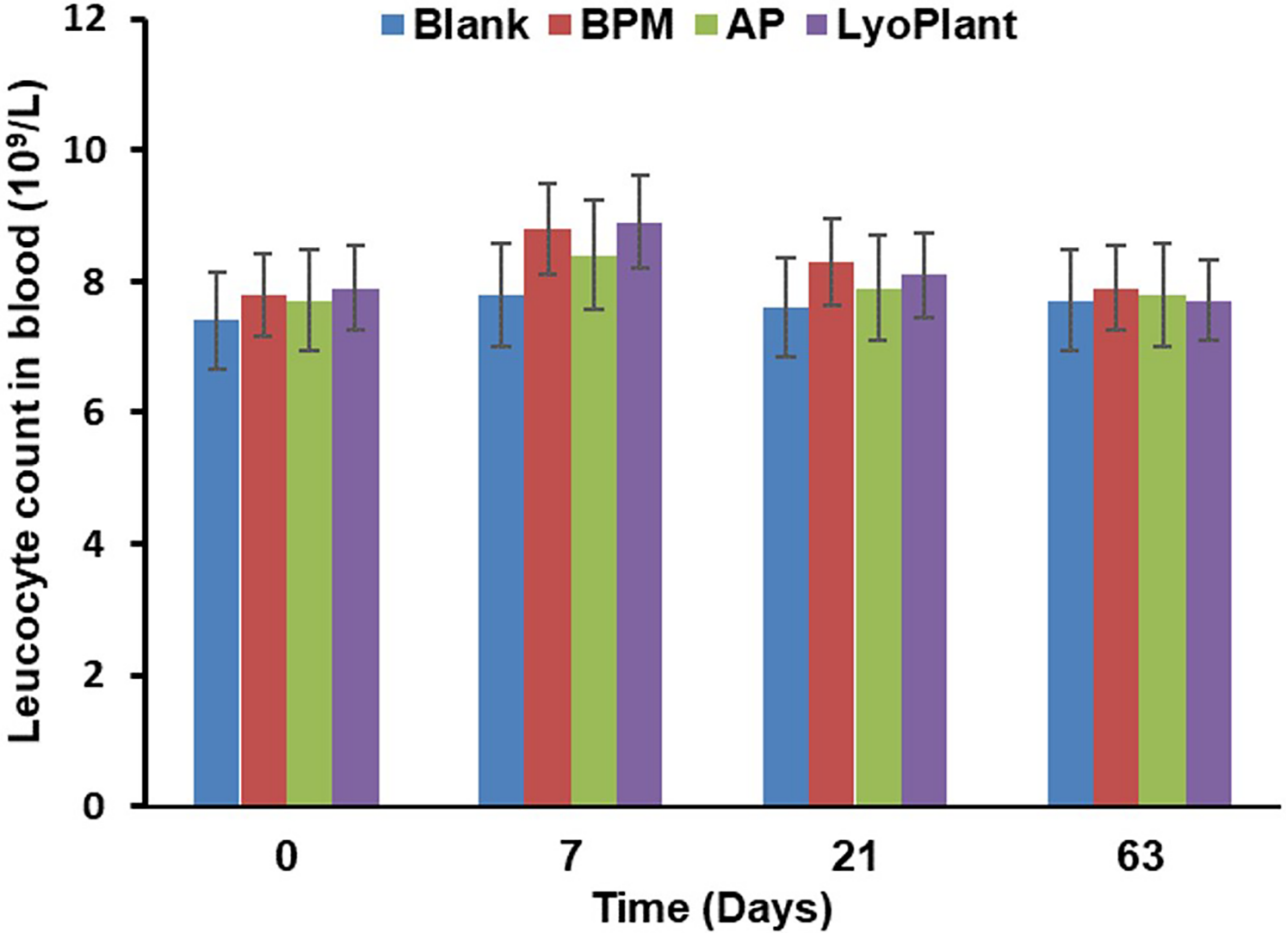
The leukocyte counts in peripheral blood after implantation of BPM, autologous periosteum (AP) and collagen membrane (lyoplant®), data are represented as the mean ± SD, (*n* = 7).

**Figure 9:**
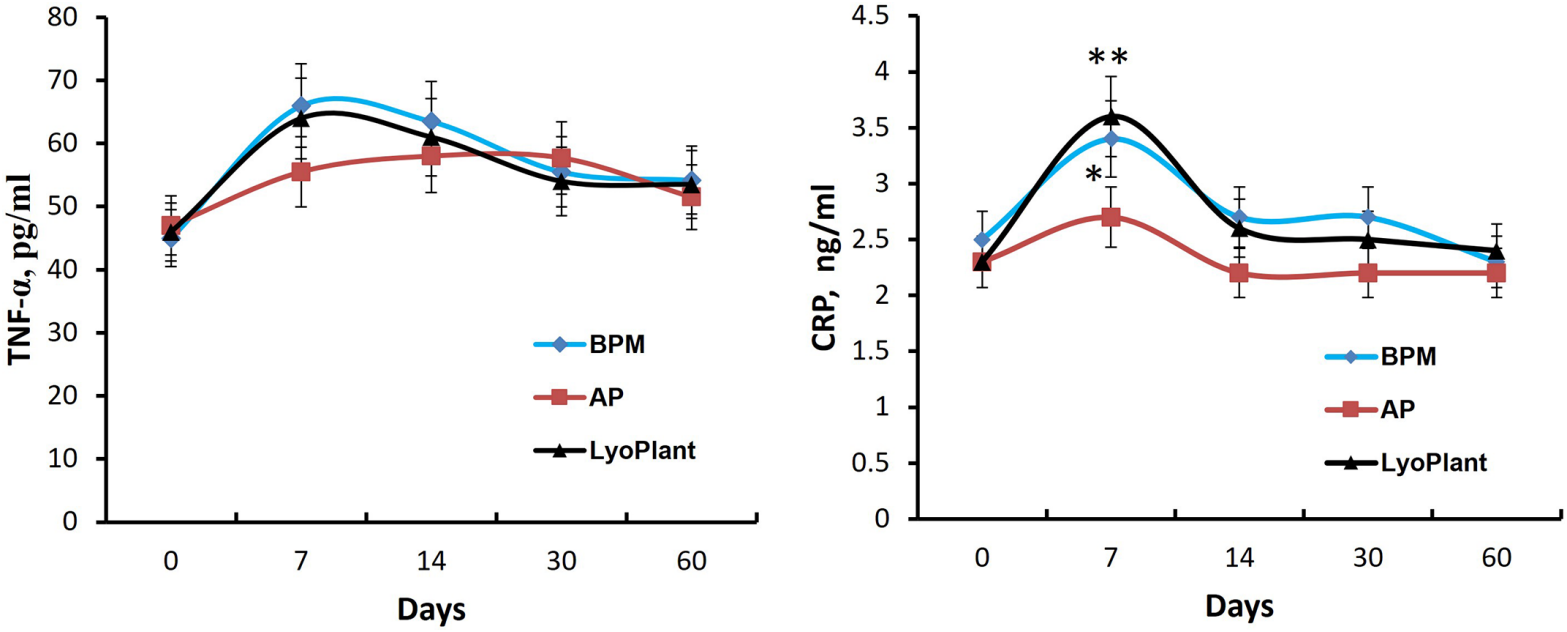
The levels of TNF-α and CRP in blood serum. Data are represented as the mean ± SD, (*n* = 5), *p < 0.05; **p < 0.01.

In order to evaluate immunological response after the implantation of the decellularized BPMs, autologous periostea, and Lyoplant® collagen membranes, the number of leucocytes and the levels of proinflammatory cytokines CRP and TNF-α in the complete blood and sera of the experimental animals were examined. The results are presented in Figures 8, 9. Regarding the leukocyte counts in the peripheral blood, insignificant differences between the experimental groups (BMP, AP, and Lyoplant®) were found at different time points.

Next, the ELISA we performed showed that the transplantation of decellularized BPMs and commercial collagen membrane samples (Lyoplant®) caused an increase in the levels of proinflammatory cytokines CRP and TNF-α in the sera of rabbits on day 7 compared with autologous periosteum group (Figure 9). However, at days 14, 30, and 60, the cytokine levels were gradually decreased to the control values.

Thus, these data indicate that BPM transplantation, as well as Lyoplant® transplantation, caused a mild inflammatory response in the experimental rabbits within 7 days which gradually normalized at day 30.

## 4 Discussion

In this study, for the first time, we examined the use of BPM as a dural substitute and compared its performance with autologous periostea and Lyoplant® collagen membranes. We studied the immunological reactions in the experimental animals and the pathomorphological changes that occurred in their implantation zones (i.e., the areas housing the dural substitutes).

For all three groups, our histological analysis showed the presence of young fibroblasts in the connective tissue membrane on the 7th day after implantation. After 7 days, we also noticed the sprouting of blood vessels. The proliferation of fibroblasts indicates that connective tissue fibers are being formed and DM is being restored. A lack of fibrous tissue formation is a disadvantage in the treatment of dural defects that require early and severe scar formation (21).

The inflammatory reactions observed in the three groups were determined to be mild, involving only a few neutrophilic–leukocyte infiltrates after 7 days. This inflammatory response is important for macrophage recruitment, which, during xenotransplantation, provides a phagocytic effect and modulates adaptive immunity (22–24); therefore, macrophage infiltration occurred in all implants. Ordikhani et al. demonstrated that the depletion of macrophages may affect the protective role of wound healing and tissue remodeling macrophages, which are required to restore homeostasis in the donor organ after surgical transplant procedures (23). However, the inflammatory reactions of the BPMs and Lyoplant® groups were a little more severe than those observed in the AP group. At the same time, insignificant differences in terms of leukocyte counts were observed at different sampling time points, indicating that no inflammatory reactions were caused by the implant or the operation.

After 21 days of implantation, regression in the inflammatory responses was observed, the fibroblasts became mature, the connective tissue fibers became denser, and proliferated meningothelial cells appeared at the border with DM. Then, at day 63, the complete fusion of the studied materials with DM was achieved.

The external surfaces of the studied materials in all groups were coated in connective tissues, and there were no fibrous adhesions or pathological changes between the dural substitutes and cerebral cortex. The existence of the connective tissues, which did not facilitate an adhesive process between DM and the brain surface, indicates a connection with the dural border cells and that passage over this border took place. Collagen and elastin fibers make up the majority of the connective tissue layer in the parietal peritoneum, giving it exceptional strength and elasticity (25).

The results of histological and macroscopic examinations performed in the study suggested that the BPMs showed a sufficient deterioration rate, i.e., one that is suitable for gathering newly formed meningothelial tissue. The thickness and density of the bovine parietal peritoneum fibers prevent resorption in the first days after use as a plastic material, and the restoration of DM itself does not occur at an accelerated pace, meaning that the gradual formation of fibrous tissue prevents adhesion to the brain surface. The same material was used in a prior investigation, which reported that decellularization minimizes the antigenic properties of the tissue and the remaining collagen matrix of the bovine parietal peritoneum is structurally similar to human collagen (26).

It needs to be accepted that rabbit DM is substantially thinner than human DM, yet this did not affect the completion of this study. Liu W. Et al. applied a rabbit model to evaluate the *in vivo* safety and dural repair performance of two different dural substitute groups and a blank control group (27). They investigated the feasibility and potential of dural regeneration by using dural substitute devices. The surgeries and implants did not cause abnormal body reactions or pyrogen reactions, and the implants caused insignificant inflammatory responses without any encapsulation or the use of antibiotics in all three groups. In addition, numerous xenografts have been investigated, as studies involving bovine or ovine pericardium (28, 29), porcine small intestinal submucosa (30, 31), and equine collagen biomatrices (32, 33) have shown that duraplasty using either allogenic or xenogeneic grafts can lead to equivalent or even favorable clinical outcomes.

In this study, we have demonstrated that decellularized BPMs are suitable biocompatible natural materials for DM repair and functional recovery, as they did not show significant adverse effects in our experiments. We suggest that the BPM may be an effective alternative option for the clinical treatment of dural defects. However, additional preclinical studies are required to further confirm decellularized BPMs' therapeutical potential for application in clinical contexts.

## Data Availability

The raw data supporting the conclusions of this article will be made available by the authors, without undue reservation.

## References

[1] Witte MB Barbul A. General principles of wound healing. Surg Clin North Am. (1997) 77(3):509–28. 10.1016/S0039-6109(05)70566-1 9194878

[2] Komotar RJ Starke RM Raper DM Anand VK Schwartz TH. Endoscopic endonasal versus open repair of anterior skull base CSF leak, meningocele, and encephalocele: a systematic review of outcomes. J Neurol Surg A Cent Eur Neurosurg. (2013) 74(4):239–50. 10.1055/s-0032-1325636 23027433

[3] Paek SH Audu PB Sperling MR Cho J Andrews DW. Reevaluation of surgery for the treatment of brain metastases: review of 208 patients with single or multiple brain metastases treated at one institution with modern neurosurgical techniques. Neurosurgery. (2005) 56(5):1021–34. 10.1227/01.NEU.0000158321.90608.BE 15854250

[4] Lawrence WT. Physiology of the acute wound. Clin Plast Surg. (1998) 25(3):321–40. 10.1016/S0094-1298(20)32467-6 9696896

[5] Lassen B Helseth E Egge A Due-Tonnessen BJ Ronning P Meling TR. Surgical mortality and selected complications in 273 consecutive craniotomiaes for intracranial tumors in pediatric patients. Neurosurgery. (2012) 70(4):936–43. 10.1227/NEU.0b013e31823bcc61 21993188

[6] Louis RG Jr Tubbs RS Mortazavi MM Shoja MM Loukas M Cohen-Gadol AA. Harvest of autologous clavipectoral fascia for use in duraplasty: cadaveric feasibility study. J Craniofac Surg. (2013) 24:619–21. 10.1097/SCS.0b013e31827c817b 23524759

[7] Caroli E Rocchi G Salvati M Delfini R. Duraplasty: our current experience. Surg Neurol. (2004) 61:55–9. 10.1016/S0090-3019(03)00524-X 14706380

[8] Lam FC Kasper E. Augmented autologous pericranium duraplasty in 100 posterior fossa surgeries-a retrospective case series. Neurosurgery. (2012) 71(2 Suppl Operative):ons302–7. 10.1227/NEU.0b013e31826a8ab0 22843136

[9] Malliti M Page P Gury C Chomette E Nataf F Roux FX. Comparison of deep wound infection rates using a synthetic dural substitute (neuro-patch) or pericranium graft for dural closure: a clinical review of 1 year. Neurosurgery. (2004) 54:599–604, discussion 603–604. 10.1227/01.NEU.0000108640.45371.1A 15028133

[10] Cappabianca P Esposito F Magro F Cavallo LM Solari D Stella L. Natura abhorret a vacuo—use of fibrin glue as a filler and sealant in neurosurgical “dead spaces”. Technical note. Acta Neurochir (Wien). (2010) 152(5):897–904. 10.1007/s00701-009-0580-2 20049488

[11] Filippi R Schwarz M Voth D Reisch R Grunert P Perneczky A. Bovine pericardium for duraplasty: clinical results in 3 patients. Neurosurg Rev. (2001) 24:103–7. 10.1007/PL00012392 11485229

[12] Vanaclocha V Saiz-Sapena N. Duraplasty with freeze dried cadaveric dura versus occipital pericranium for chiari type I malformation: comparative study. Acta Neurochir (Wien). (1997) 139:112–9. 10.1007/BF02747190 9088368

[13] Brown BN Badylak SF. Extracellular matrix as an inductive scaffold for functional tissue reconstruction. Transl Res. (2014) 163:268–85. 10.1016/j.trsl.2013.11.003 24291155 PMC4203714

[14] Abugaliyev KR Ogay VB Danlybayeva GA Silayev DV. Biologic Wound Dressing. Eurasian Patent Organization 2018. Eurasian Patent for Invention No. 201400992, EAPO Bulletin No. 11 (2015).

[15] Tuleubayev B Ogay V Anapiya B Zhylkibayev A Saginova D Koshanova A Therapeutic treatment of 2A grade burns with decellularized bovine peritoneum as a xenograft: multicenter randomized clinical trial. Medicina (Kaunas). (2022) 58(6):819. 10.3390/medicina58060819 35744082 PMC9230981

[16] Tuleubayev BE Anapiya BB Kurmangaliyev YT Abugaliyev KR. Successful treatment outcomes for partial thickness burns by innovative bovine peritoneum dressing. Plast Reconstr Surg Glob Open. (2022) 10(2):e4150. 10.1097/GOX.0000000000004150 35233339 PMC8878626

[17] Ogay VB Danlybayeva GA Abugaliyev KR. Biological dressing for the Treatment of Burns and Wounds. Patent for invention of the Republic of Kazakhstan №30382, Bulletin No 9 (2015).

[18] Crapo PM Gilbert TW Badylak SF. An overview of tissue and whole organ decellularization processes. Biomaterials. (2011) 32(12):3233–43. 10.1016/j.biomaterials.2011.01.057 21296410 PMC3084613

[19] Keane TJ Londono R Carey RM Carruthers CA Reing JE Dearth CL Preparation and characterization of a biologic scaffold from esophageal mucosa. Biomaterials. (2013) 34(28):6729–37. 10.1016/j.biomaterials.2013.05.052 23777917 PMC3727430

[20] Keane TJ Swinehart IT Badylak SF. Methods of tissue decellularization used for preparation of biologic scaffolds and in vivo relevance. Methods. (2015) 84:25–34. 10.1016/j.ymeth.2015.03.005 25791470

[21] Tachibana E Saito K Fukuta K Yoshida J. Evaluation of the healing process after dural reconstruction achieved using a free fascial graft. J Neurosurg. (2002) 96(2):280–6. 10.3171/jns.2002.96.2.0280 11838802

[22] Hu M Hawthorne WJ Yi S O’Connell PJ. Cellular immune responses in islet xenograft rejection. Front Immunol. (2022) 13:893985. 10.3389/fimmu.2022.893985 35874735 PMC9300897

[23] Ochando J Kwan WH Ginhoux F Hutchinson JA Hashimoto D Collin M. The mononuclear phagocyte system in organ transplantation. Am J Transplant. (2016) 16(4):1053–69. 10.1111/ajt.13627 26602545

[24] Ordikhani F Pothula V Sanchez-Tarjuelo R Jordan S Ochando J. Macrophages in organ transplantation. Front Immunol. (2020) 11:582939. 10.3389/fimmu.2020.582939 33329555 PMC7734247

[25] Holt D Agnello KA. Peritoneum. In: Langley-Hobbs SJ Demetriou JL Ladlow JF, editors. Feline Soft Tissue and General Surgery. New York, NY: Elsevier Science Incste. (2014). p. 281.

[26] Wong ML Griffiths LG. Immunogenicity in xenogeneic scaffold generation: antigen removal vs. decellularization. Acta Biomater. (2014) 10:1806–16. 10.1016/j.actbio.2014.01.028 24486910 PMC3976714

[27] Liu W Wang X Su J Jiang Q Wang J Xu Y In vivo evaluation of fibrous collagen dura substitutes. Front Bioeng Biotechnol. (2021) 9:628129. 10.3389/fbioe.2021.628129 33681163 PMC7930396

[28] Anson JA Marchand EP. Bovine pericardium for dural grafts: clinical results in 35 patients. Neurosurgery. (1996) 39(4):764–8. 10.1097/00006123-199610000-00025 8880771

[29] Parizek J Husek Z Mericka P Tera J Nemecek S Spacek J Ovine pericardium: a new material for duraplasty. J Neurosurg. (1996) 84(3):508–13. 10.3171/jns.1996.84.3.0508 8609566

[30] Bejjani GK Zabramski J. Safety and efficacy of the porcine small intestinal submucosa dural substitute: results of a prospective multicenter study and literature review. J Neurosurg. (2007) 106(6):1028–33. 10.3171/jns.2007.106.6.1028 17564175

[31] Cobb MA Badylak SF Janas W Simmons-Byrd A Boop FA. Porcine small intestinal submucosa as a dural substitute. Surg Neurol. (1999) 51(1):99–104. 10.1016/S0090-3019(97)00475-8 9952131

[32] Biroli F Esposito F Fusco M Bani GG Signorelli A de Divitiis O Novel equine collagen-only dural substitute. Operative Neurosurg. (2008) 62(3):273–4. 10.1227/01.neu.0000317404.31336.69 18424997

[33] Gazzeri R Neroni M Alfieri A Galarza M Faiola A Esposito S Transparent equine collagen biomatrix as dural repair. A prospective clinical study. Acta Neurochir. (2009) 151(5):537–43. 10.1007/s00701-009-0290-9 19337680

